# Detection and Quantification of SARS-CoV-2 Receptor Binding Domain Neutralization by a Sensitive Competitive ELISA Assay

**DOI:** 10.3390/vaccines9121493

**Published:** 2021-12-16

**Authors:** Ahmed O. Shalash, Armira Azuar, Harrison Y. R. Madge, Naphak Modhiran, Alberto A. Amarilla, Benjamin Liang, Alexander A. Khromykh, Daniel Watterson, Paul R. Young, Istvan Toth, Mariusz Skwarczynski

**Affiliations:** 1 School of Chemistry and Molecular Biosciences, The University of Queensland, St. Lucia, QLD 4072, Australia; a.shalash@uq.net.au (A.O.S.); armira.azuar@uq.net.au (A.A.); harrison.madge@uq.net.au (H.Y.R.M.); n.modhiran@uq.edu.au (N.M.); a.amarillaortiz@uq.edu.au (A.A.A.); benjaminjianwen.liang@uq.net.au (B.L.); alexander.khromykh@uq.edu.au (A.A.K.); d.watterson@uq.edu.au (D.W.); p.young@uq.edu.au (P.R.Y.); i.toth@uq.edu.au (I.T.); 2 Australian Institute for Bioengineering and Nanotechnology, The University of Queensland, St. Lucia, QLD 4072, Australia; 3 Institute for Molecular Bioscience, The University of Queensland, St. Lucia, QLD 4072, Australia; 4 School of Pharmacy, The University of Queensland, Woolloongabba, QLD 4102, Australia

**Keywords:** SARS-CoV-2, receptor binding domain, spike protein, binding inhibition assay, neutralization assay, competitive ELISA, validation, high throughput

## Abstract

This protocol describes an ELISA-based procedure for accurate measurement of SARS-CoV-2 spike protein-receptor binding domain (RBD) neutralization efficacy by murine immune serum. The procedure requires a small amount of S-protein/RBD and angiotensin converting enzyme-2 (ACE2). A high-throughput, simple ELISA technique is employed. Plate-coated-RBDs are allowed to interact with the serum, then soluble ACE2 is added, followed by secondary antibodies and substrate. The key steps in this procedure include (1) serum heat treatment to prevent non-specific interactions, (2) proper use of blank controls to detect side reactions and eliminate secondary antibody cross-reactivity, (3) the addition of an optimal amount of saturating ACE2 to maximize sensitivity and prevent non-competitive co-occurrence of RBD-ACE2 binding and neutralization, and (4) mechanistically derived neutralization calculation using a calibration curve. Even manually, the protocol can be completed in 16 h for >30 serum samples; this includes the 7.5 h of incubation time. This automatable, high-throughput, competitive ELISA assay can screen a large number of sera, and does not require sterile conditions or special containment measures, as live viruses are not employed. In comparison to the ‘gold standard’ assays (virus neutralization titers (VNT) or plaque reduction neutralization titers (PRNT)), which are laborious and time consuming and require special containment measures due to their use of live viruses. This simple, alternative neutralization efficacy assay can be a great asset for initial vaccine development stages. The assay successfully passed conventional validation parameters (sensitivity, specificity, precision, and accuracy) and results with moderately neutralizing murine sera correlated with VNT assay results (R^2^ = 0.975, *n* = 25), demonstrating high sensitivity.

## 1. Introduction

Infection by SARS-CoV-2 virus (SARS-2) occurs mainly through inhalation of virus-laden respiratory droplets [1]. When the virus reaches the lower lung airways, ACE2-expressing pneumocytes-II epithelial cells take up SARS-2 through interaction between host cell ACE2 receptors and the viral spike-protein (S-protein) [2,3,4]. Thus, neutralization is mainly achieved by antibodies (Abs) that interrupt the binding of ACE2 receptors and SARS-CoV-2 S-protein receptor binding domain (RBD) [3,4,5]. To fast-track the development of effective vaccines against SARS-CoV-2, surrogate high-throughput efficacy tests are urgently needed. Neutralization efficacy screening of vaccine candidates can be expedited by abstracting/distilling neutralization evaluation into a binding study between ACE2 and S-protein, or its RBD domain, in the presence or absence of vaccinated animal or human immune serum [6,7,8].

ELISA is a simple, high-throughput serological assay, in which antigens are coated onto disposable microtiter plates, and antigen-specific Abs from immune serum are allowed to bind to coated antigens. Binding is then detected by a chromogen-labeled secondary (2^ry^) Ab/probe [9,10,11]. ELISA is not only used to evaluate antigen-specific immune responses, it is also used to study binding affinity and kinetics, singularly or competitively, to extract meaningful binding values, e.g., the binding dissociation constant (*K_D_*) or binding extent [6,7,8]. Competitive ELISA (cELISA) demonstrates binding competition between two proteins that separately bind to the same location on an antigen [9,10,11]. However, when both competitor proteins are present, the protein with the more favorable binding characteristics and affinity binds preferentially and inhibits the competitor protein’s binding, as they sterically clash with each other.

Plaque reduction neutralization (PRNT) and virus neutralization (VNT) cell-based assays are considered to be the ‘gold standards’ for evaluating the neutralization efficacy against viruses [5,6,12]. Because these assays simulate an in vivo environment, with host cells and viruses present in biological media, they are capable of demonstrating the efficacy of different immune sera or antivirals, and their results correlate well with in vivo protection against infection challenge [13]. However, cell-based assays are not feasible for high-throughput screening of large numbers of sera, as they are laborious, require special biosafety containment, and are subject to long culturing and incubation times for host cells with live infectious viruses [14,15]. Cell-based surrogate neutralization assays are also relative in their assessment of neutralization efficacy: outcomes may vary depending on the assay set-up, e.g., seeded cell counts, ACE2 expression levels, and virus dose, even when the same assay is used. Therefore, external standards, for example, known neutralizing Abs, are often used with each assay procedure to allow for comparison of outcomes. Despite variations, when proper controls and standard neutralizing Abs in cell-based assays are used, the assays generally correlate well with each other and with the protection afforded in infection challenge [5,16,17]. It is important to note that cELISA is not intended to replace PRNT or VNT assays, as they are more bio-relevant, i.e., they more closely represent natural infection at the cellular level in complex biological environments. However, cELISA assays are well-suited to screen a large number of sera in a short time, can be fully-automated, and follow a simple, straightforward procedure, which does not require sterile conditions, special containment, or time-consuming cell culturing. The cELISA strategy is a very efficient tool to fill the gap between initial screening of large numbers of immune sera from animals immunized with various vaccines and pinpoint highly effective vaccine candidates in a short time. Thus, we aimed to develop an assay that correlates with the well-established gold standard neutralization assessment assays for accurate, quantitative determination of neutralization efficacy/potential.

## 2. Comparison to Other Competitive ELISA Assays

**Figure 1:**
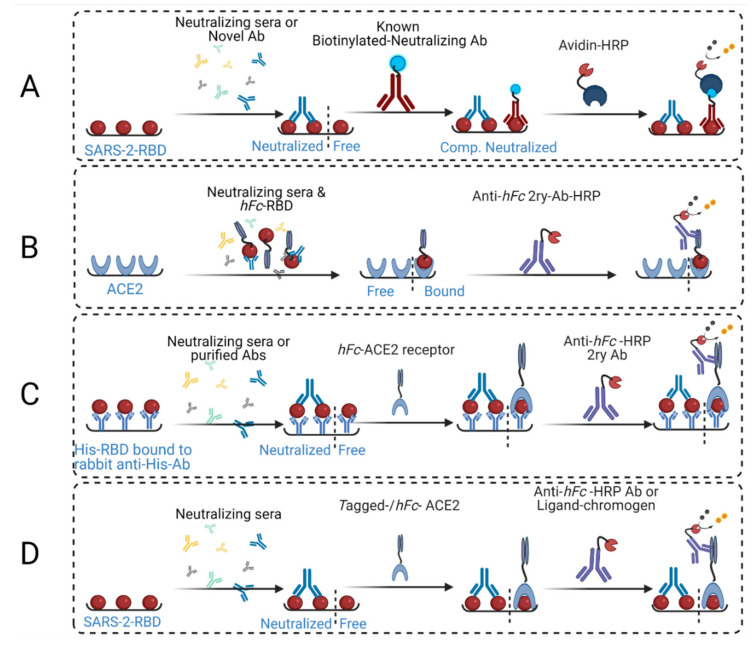
Schematic representation of various reported assay strategies. (**A**) This strategy involves coating ELISA plates with SARS-2-RBD, employing neutralizing sera or unknown Abs, applying known competing biotinylated neutralizing Abs, then adding chromogen-streptavidin followed by substrate for color generation and detection via plate common readers. (**B**) This strategy involves coating ELISA plates with ACE2, then employing a mixture of neutralizing sera and human IgG-Fc-RBD conjugate (*hFc*-RBD), followed by anti-human IgG-Fc-HRP-conjugated secondary Abs (Anti-*hFc*-2^ry^ Ab-HRP) and substrate. (**C**) This strategy utilizes the capture ELISA principle, where anti-histidine rabbit Abs are coated onto the plates, followed by capture of the applied His-tagged RBD. Neutralizing sera or purified antibodies are applied, followed by human IgG-Fc-conjugated ACE2 (*hFc*-Ace2), then anti-*hFc*-2^ry^ Ab-HRP and substrate. (**D**) Our strategy, which is reported herein, involves coating the ELISA plates with RBD, applying neutralizing sera and *hFc*-ACE2 (or biotin-tagged ACE2), then adding anti-*hFc*-2^ry^ Ab-HRP (or chromogen-streptavidin), respectively, with substrate for color generation and detection.

There are several configurations of cELISA assays (Figure 1), reflecting the variation in reagents and protein concentrations and total applied quantities, order of addition, and procedures. In early cELISA [18,19,20,21] designs, wells were coated with RBD, followed by the addition of immune serum, but ACE2 as an RBD-binding competitor protein was not employed (Figure 1A). Instead, a known nAb was applied afterwards to determine whether the newly investigated serum, or purified Abs, competed for the same neutralizing epitope. However, protective antibodies (Abs) may independently target different neutralizing epitopes, and thus, they do not exhibit competitive binding to the known nAb. Furthermore, while Abs that bind competitively to the same epitope sterically clash with each other, this does not prove inhibition of ACE2 binding, as critical ACE2-binding residues within the shared epitope may not be challenged by the tested Ab or serum. A neutralizing epitope was recently shown to overlap with an infection-enhancing epitope in a SARS-CoV pre-clinical study on macaques [22], and thus, steric clashing of antibodies against these epitopes does not guarantee ACE2-RBD binding neutralization.

Eventually, ACE2-coated plates were employed, and a mixture of RBD and neutralizing serum was added to the plates [17,18] (Figure 1B). The advantage of this configuration is that it requires fewer processing steps. However, it was difficult to use controls, as RBD-neutralizing Ab binding inhibited ACE2-RBD binding. Thus, bound, or excess unbound RBDs, were washed away with the serum before 2^ry^ Abs were added. Therefore, serum interaction with RBD, whether as non-specifically bound or excess unbound molecules, could not be detected or confirmed by controls. This assay strategy also requires manipulation of serum/neutralizing Ab concentration, serum to RBD ratio, and mixing with RBD before it is added to the ACE2-coated plates. Thus, neutralization (%) can be easily evaluated only at very few high serum concentration values, as RBD and sera have to be mixed first before addition to the wells. In addition, nAb/RBD mixing time is critical, as binding kinetics influence the extent/percentage of RBD-nAb binding over time [23,24]; as such, the mixing/incubation time requires further investigation in assay strategy B. In contrast, standard ELISA procedures guarantee antigen-specific binding to reach true equilibrium, even for viscous sera, after 1–2 h of incubation. Lastly, in He Y. et al., the concentration of purified Abs that were employed in this assay strategy were high (50 µg/mL). This would have affected binding affinity and might even have allowed for the binding of weakly neutralizing Abs [23].

Recently, a more complicated RBD/S-protein coating was employed. Rabbit anti-histidine Abs (1 µg/mL) was first coated on microtiter plates, then His_6_-RBD or His_6_-S-protein (10 µg/mL) solution was added to the wells in a similar manner to sandwich ELISA (Figure 1C) [25]. Sera or purified Abs were subsequently added to neutralize indirectly-coated RBD or S-protein. Human IgG-Fc-conjugated ACE2 (*hFc*-ACE2) was applied, followed by 2^ry^ Abs and substrate. This strategy is similar to our approach (Figure 1D) [8,25], although the initial anti-His coating step is not included in our strategy since RBD or S-protein binds well to ACE2 when coated directly onto the plate [8]; however, no additional controls were employed, in strategy (C), to determine the amount of indirectly bound S-protein. Assays that use anti-His coating require extra time and effort due to addition of competitor ACE2 protein, which is generally longer than standard ELISA. This protocol used an unknown quantity of S-protein, which was indirectly coated on the plates, and suboptimal amounts of ACE2 (0.1 µg/mL). Low quantities of ACE2 (below saturation) could result in the presence of unbound S-protein, i.e., neither bound by ACE2 nor by neutralizing Abs. This would influence total optical density (OD) readings, because neutralization and ACE2 binding could occur at the same time. Therefore, in our assay strategy, we used enough ACE2 to reach saturation to ensure the assay’s sensitivity for neutralization detection.

**Figure 2:**
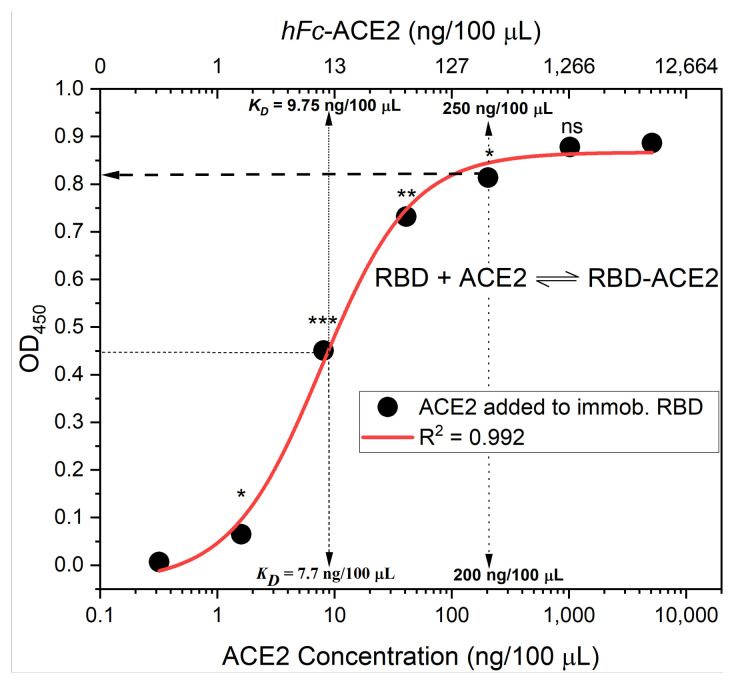
The binding affinity of ACE2 added to immobilized RBD. RBD (50 ng/100 µL per well) was immobilized on ELISA plates, then ACE2 solutions with various concentrations were added to the immobilized RBD. Bound ACE2 and saturation were determined from the OD_450_ signal and fitted to a four-parameter logistic function, providing an R^2^ = 0.99 and a binding affinity dissociation constant of *K_D_* = 10 ng/100 µL, as determined at 50% of the maximum signal reading. The highest reading that resulted in significant OD increase corresponded to 250 ng/100 µL of *hFc*-ACE2. Therefore, a 5:1 concentration ratio of *hFc*-ACE2 to RBD (or 200 ng/100 µL, 4:1 ratio of ACE2/biotinylated-ACE2) is required for saturation with a statistically significant maximum OD signal (*p* < 0.05). This corresponds to an equimolar ratio between the two proteins that achieves a 1:1 binding ratio. Asterisks represent the statistical significance level of each data point (compared to the preceding data point) using the student *t*-test, * *p* ≤ 0.05; ** *p* < 0.01; *** *p* < 0.001; ns is non-significant, *p* > 0.05.

Currently, the most common cELISA strategy [6,7], which has also been reported by our group [8], involves allowing RBD- or S-protein-coated plates to interact with immune sera, i.e., become neutralized. Thereafter, *hFc*-ACE2 (or biotinylated-ACE2, for testing human sera) is added, followed by anti-*hFc* 2^ry^ Ab-HRP (or streptavidin-HRP for human serum) and substrate. This strategy involves sequential addition of binding proteins (serum antibodies, 2^ry^ antibody, and ACE2), and thus, facilitates full use of controls. This enables the detection of non-specific interactions, e.g., between RBD and serum proteins, such as complement proteins and detects cross-reactivity of 2^ry^ Abs with primary/murine Abs or residual serum proteins, e.g., by including blank ACE2 control. In the reported tests, 100 ng of RBD (50 µL of 1 µg/mL solution) was used for plate coating; however, only 50 ng of ACE2 (100 µL of 1 µg/mL solution) was added. Despite that, a four- to five-fold increase in ACE2 quantity was required to achieve equimolar saturation of coated RBD (Figure 1D). Through development of our assay, we found that 50 ng of directly coated RBD provided a sufficient OD_450_ signal, even when it was partially bound to ACE2. The resulting signal was within the linear range of Beer–Lambert’s Law, and protein sparing as well, because 200–220 ng of ACE2 is needed, per well, to saturate coated-RBD and result in an OD_450_ signal (plateau) (Figure 2).

All reported assay strategies have assumed linear calculations, as the signal OD value is divided by a blank and then subtracted from 100% to yield percent neutralization. This assumes linear RBD-ACE2 binding or competition, as well as full ACE2 saturation at low serum concentration [6] or in the absence of serum [7]. However, all of these assumptions are incorrect (Figure 2). While Abe et al. [6] employed a different calculation approach, they used area under the curve (AUC) displacement as an independent neutralization assessment parameter in their cELISA as an alternative relative neutralization measurement.

While potently neutralizing sera can maintain neutralization at dilutions of up to 1/200–1/1000, we found significant positive interference (>10% OD_450_ of non-neutralizing control) in assays with high serum concentrations (>2%) if the serum was not heat-treated (not shown). The correlation between neutralization results of cELISA and pseudovirion cell entry assay was R^2^ = 0.76, but this dropped to 0.6 between cELISA and PRNT assays (VNT equivalent), potentially due to serum-associated interference [6]. Ultimately, none of the reported assay methods or protocols employed heat inactivation of the sera, even at high concertation’s, nor did they employ calibration curves to evaluate diluted immune serum signals. We found that these two steps were essential for accurate determination of the neutralization efficacy of immune sera.

## 3. Development of cELISA Assay

Several critical aspects of cELISA assay development were not sufficiently considered in previous assay designs. These include (a) the ACE2 solution concentration (>*K_D_*) employed, (b) the total amount of ACE2 applied (to saturate bound RBD), (c) the use of a non-linear calibration curve for calculations (considering the inaccuracies introduced from a linear assumption), (d) maximizing the sensitivity of the OD signal through adjustment of the RBD quantity, while maintaining the optimum RBD/ACE2 ratio, (e) ensuring assay specificity by eliminating non-specific binding interference of high serum concentrations to added ACE2 or 2^ry^ Abs, and (f) ensuring that the conventional validation parameters conformed to standard assay specifications by evaluating accuracy, precision, quality of non-linear fit, specificity, and sensitivity (i.e., detection and quantification limits). In order to ensure that the binding equilibrium is shifted towards ACE2 binding of RBD, unless nAbs are present, the concentration of added ACE2 solution must exceed the *K_D_* of ACE2-RBD binding for a given coated amount of RBD. Otherwise, even in presence of ACE2, minimal binding would be expected, or the equilibrium could shift towards RBD binding to a weakly neutralizing competitor Ab, instead of ACE2. The minimum ACE2 concentration required for favorable binding equilibrium to coated RBD at 50 ng/100 µL per well was *K_D_* = 10 ng/100 µL (0.083 nM) for Fc-ACE2. However, this still does not ensure the saturation of all coated RBD with ACE2. Therefore, the concentration should exceed the *K_D_* and the binding extent should be near saturation, i.e., all coated and accessible RBDs (26.6 kDa) should bind to Fc-ACE2 (120 kDa). Thus, an equimolar ratio per well of both proteins may be employed (RBD/ACE2 ratio of 0.23). Ultimately, Fc-ACE2 total mass per well should be four- to five-fold the amount of coated RBD to ensure maximum coverage/saturation. We employed this ratio in our binding curve (Figure 2), as this was the maximum amount of ACE2 that resulted in a significant increase in OD_450_ signal.

When using lower amounts of RBD, which has commonly been done, accessible, unbound RBDs are available in the presence of a suboptimal amount of ACE2, resulting in simultaneous, non-competitive Ab-neutralization and ACE2/RBD binding. However, due to the non-linearity of binding approaching saturation, changes in OD readings are very minor (at the plateau), while the amount of ACE2 still increases. Therefore, this may significantly influence readings near the saturation point. To solve this issue, standard neutralizing Abs with known neutralization efficacies (*N*_50_ or *IC*_50_) must be employed, or a saturating amount of ACE2 must be used. Otherwise, significant differences between the OD readings (signal replicates) from different amounts of bound ACE2 may be observed, reducing the accuracy of the assay. Binding equilibria are non-linear and follow a sigmoidal pattern (Figure 2), even in the absence of a competitor probe, e.g., neutralizing Abs. Therefore, accounting for neutralization by the missing or unbound ACE2 using only the difference between OD readings and the blank (absent serum) plate wells assumes a linear relationship between bound ACE2 (%) and OD readings (Figure 2), which is grossly inaccurate. This linear relationship assumption in the calculation is, unfortunately, common across all reported forms of the cELISA assay, and this is likely one of the main reasons for the technique’s limited correlation with cell-based assays.

The OD_450_ readings are relative for every measurement, as they depend on substrate-enzyme type, properties, concentration, and reaction time. Thus, a calibration curve or external standard is required for each measurement. This has also, unfortunately, not been employed in any of the reported cELISA assays, as they falsely assumed that the highest reading corresponded to 100% bound ACE2, and that values less than this maximum represented unbound ACE2, i.e., full neutralization of immobilized RBD in a linear fashion. This is incorrect due to a number of factors, including positive interference from non-specific binding. Therefore, a calibration curve must be established to quantify the amount of bound or free ACE2 as an external standard for the determination of neutralization (%).

The total amount of coated RBD should be high enough to obtain a clear OD readout signal, thus increasing test sensitivity. However, it should not be high enough to exceed the Beer–Lambert cut-off of 0.9 absorbance unit in order to avoid convoluting the calculations further with an additional non-linear component. The immune serum must not interact with any of the ingredients, so as not to impart significant interference. This last point is very important, as we found that the serum non-specifically binds to ACE2 and/or 2^ry^ Abs at concentrations exceeding 2–5%, even with extensive plate washing. We found in early development of the assay that OD readings from sera wells at various serum concentrations (RBD + 5–20% serum + ACE2 + 2^ry^ Abs) could be twice as high as maximum OD readings from blank serum wells in the calibration curve (100% RBD + ACE2 + 2^ry^ Ab) that should have maximum theoretical OD signal. This shows that serum complement activation at high concentrations non-specifically traps ACE2. Similarly, irrelevant serum from a PBS-immunized group of mice (*n* = 5) at a high concentration (2–20%) was also found to result in a low but significantly high OD reading interference in blank ACE2 control (RBD + 2–20% serum + 2^ry^ Ab) compared to background OD readings, despite the absence of antigen-specific Abs and ACE2. This shows that complement activation could also trap 2^ry^ Abs. Therefore, as commonly used in biotechnology techniques, heat inactivation of the serum was conducted to eliminate this non-specific binding/trapping effect. Following heat inactivation, the background interference was minimal (<5% of total OD), even at the highest employed serum concentrations (1/10 dilution), thus increasing assay specificity and sensitivity to neutralization detection and accurate quantification. Another alternative technique involves purifying and isolating RBD-specific IgGs to evaluate them directly in the assay. While purified Abs are easier to evaluate, serum testing requires these precautions and a number of controls to ensure that no significant interference or non-specific interactions are present. Moreover, conventional validation parameters and compendial limits are also important to investigate, even if their boundaries are often less strict for immunological and biological techniques because of their inherent variability. Important validation parameters include accuracy, specificity, precision, sensitivity, and non-linear calibration curve fit quality. Finally, serum assay results should correlate and conform to gold standard cell-based neutralization assays to prove validity and usefulness as an alternative high-throughput screening technique for neutralization efficacy determination.

The relationship between binding affinity/extent and added total substrate/binder protein concentration kinetics follows a non-linear sigmoidal pattern “exponential rise to plateau”. Thus, the simplest mathematical function to represent binding is the Michaelis–Menten adsorption equation. Alternatively, the more flexible four-parameter logistic sigmoidal function, which results in a better fit with more complex binding affinity curves, could also be used (Equation (1)):

(1) $$y=\frac{A_{1}-A_{2}}{1+{\left(x/x_{o}\right)}^{p}}+A_{2}$$

where A_1_ and A_2_ are the minimum and maximum measured/found OD signals that corresponds to the amount of bound ACE2, p is the power exponent representing the growth rate of the signal as the binder protein concentration increases, and x_o_ is the x-axis ACE2 concentration value at 50% binding, i.e., half-height of the maximum OD signal (Figure 2).

The amount of bound ACE2 can only be determined via constructing and interpolating an ACE2-RBD binding calibration curve. Employing ACE2 concentrations below saturation will reduce test sensitivity to detect neutralization. Applying excess ACE2 beyond saturation will not change the signal at the plateau (Figure 2), thus wasting expensive ACE2 protein, which could be disrupting to the binding equilibria. Therefore, the ideal amount of ACE2 is at the beginning of the RBD/ACE2 binding plateau, where the differences in OD-readings beyond which becomes non-significant statistically, e.g., 250 ng/100 µL concentration point in Figure 2. This can be calculated experimentally or provided from a supplier’s RBD certificate of analysis effective 50% binding concentration/molar ratio to RBD value, if available. Finally, the OD-reading should be adjusted to lie within a linear absorbance range of 0.1 to 0.9, in accordance with Beer–Lambert’s Law, as greater values will impart non-linearity to the response and unnecessarily complicate the calculations.

**Figure 3:**
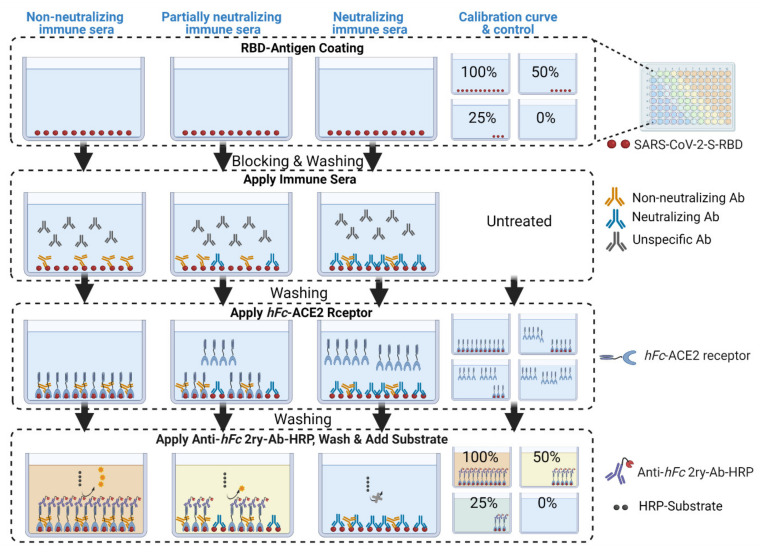
Schematic diagram depicting the cELISA processing steps and expected outcomes. RBD antigen is coated onto sample plates and, in different amounts, onto a calibration curve plate. The wells are blocked with 2% BSA solution to prevent further non-specific binding.

In our procedure, we took into account all eight of the issues mentioned above. We conducted our cELISA assays using the configuration presented in Figure 1D and Figure 3 on several immune murine sera to evaluate neutralization efficacy. We interrogated the procedure using conventional assay method development validation parameters, such as non-linear calibration curve fit and significance, precision, accuracy, specificity, and sensitivity in terms of the limit of quantification and detection of neutralization. Lastly, we conducted a standard cell-based neutralization efficacy assay (VNT assay) to compare and correlate the neutralization efficacies of both tests.

## 4. Evaluation of Conventional Validation Parameters

We adopted rigorous compendial analytical validation parameters to qualify the neutralization detection and quantification capabilities of this assay. Non-linear calibration curve fit quality is important to confidently determine and interpolate the neutralization values of each well at each serial serum dilution, as demonstrated in the procedure. Goodness of fit was evaluated by correlation coefficient value (R^2^ > 0.95). Furthermore, standard deviations between each two successive mean-bound ACE2 amounts in the calibration curves (*n* = 5) was statistically significant (*p* < 0.05, paired student *t*-test). This ensures that there is no overlap between two successive neutralization values. Our procedural set-up confirms significant differences and excellent fitting of logistic function. Our 4P logistic fit of the calibration curve gave correlation coefficient (R^2^) values that were often in the range of 0.98 to 0.99. Precision or repeatability evaluation was conducted on four different serum samples with various neutralization efficiencies through determination of the 50% neutralization (*N*_50_) and percent neutralization extent (*N*%) values. *N*% is the percent reduction/neutralization of ACE2-RBD binding at a given serum dilution, and *N*_50_ is the serum dilution (or antibody concentration) responsible for reducing/neutralizing ACE2-RBD binding to 50%. The *N*_50_ and *N*% for each serum (at different dilutions) had coefficients of variance below <5%. Since the calibration curve should simulate neutralization conditions to different extents (*N*%), we immobilized different amounts of RBD on ELISA plates. This is because when neutralization occurs, the RBDs become inaccessible to ACE2, i.e., less RBDs are available, so we deliberately reduced the amount of coated RBDs.

Accuracy was determined by adding irrelevant serum (which does not bind to RBD) to the calibration curve with various coated-RBD concentrations, ranging from 2.5 to 50 ng/100 µL/well. These corresponded to 95% to 0% of neutralization, respectively. The blank (subtracted) calibration curve OD_450_ readings (in the presence of irrelevant murine serum) remained within ±5% of the labeled bound ACE2 (%) standard value (in absence of the serum), without statistically significant differences (student *t*-test, *p* > 0.05, *n* = 4). Specificity was evaluated by testing the neutralization potential of a non-neutralizing irrelevant serum sample and determining whether it had a significant neutralizing response at any dilution. The non-neutralizing serum had no detectable neutralization at the highest concentration and the level of interference was below 2%, which was attributed to acceptable random error. The sensitivity of the assay was evaluated by its capacity for quantitative detection of neutralizing sera. The neutralization detection limit was calculated from the mean background OD value plus three standard deviations (OD_450_ = 0.056), which corresponded to 0.3% bound ACE2. The quantitation limit was the mean background OD value plus 10 standard deviations (OD_450_ = 0.083), which corresponded to 7% bound ACE2. These represent very sensitive detection and quantification limits (Figure 1D), as the lowest OD signals result from the lowest amount of bound *hFc*-ACE2 (high neutralization extent), and thus, in turn, they have the lowest amount of bound 2^ry^ Abs, which are responsible for the color change.

## 5. Virus Neutralization Assay: Orthogonal Validation

**Figure 4:**
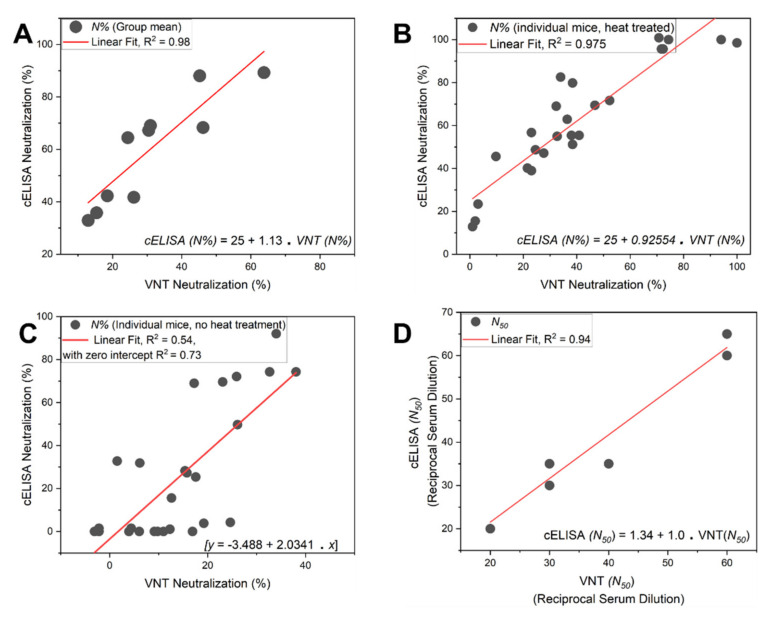
The correlated data belong to six groups of mice, comprising 24 different weakly-to-moderately neutralizing murine sera at different dilutions: (**A**) the correlation between combined group mean neutralization (%) values from the VNT assay and heat-inactivated sera cELISA assay was R^2^ = 0.98, *n* = 10; (**B**) the correlation between individual mouse immune serum neutralization (%) values from the VNT assay and heat treated serum cELISA assay was R^2^ = 0.975, *n* = 25; (**C**) the correlation between individual mouse immune serum neutralization (%) values from the VNT assay and untreated serum cELISA assay was R^2^ = 0.54, *n* = 25; (**D**) the correlation between reciprocal serum dilutions that corresponded to 50% neutralization efficacy (*N*_50_) between both assays (VNT and heat-treated serum cELISA) using heat-inactivated weak-to-moderately neutralizing murine sera profiles that were within the detectable range of both assays.

Twenty-five individual murine immune sera were tested (*n* = 25) for neutralization using both VNT and cELISA assays. Mice were immunized with various SARS-CoV-2 vaccines or a PBS negative control. Group mean neutralization extent (%) was calculated using both assays by averaging the neutralization efficacy (%) for each serum dilution (1/20, 1/40, or 1/80) (Figure 4A). cELISA assays were additionally conducted with and without heat treatment (Figure 4B,C). Finally, the serum neutralization profile and dilution value corresponding to *N*_50_ were determined by both assays and compared (Figure 4D). The calculation protocol for the cELISA technique is provided in the detailed protocol.

VNT assays were conducted as described previously (Figures S1 and S2) [26,27]. Briefly, overlay medium, block buffer, and polysorbate/phosphate saline (wash buffer) were prepared. The primary probes (mouse sera) were heat inactivated. Vero E6 cells were cultured in DMEM medium. SARS-2 virus isolate (QLD02, GISAID accession EPI_ISL_407896, provided by Queensland Health Forensic and Scientific Services, Queensland Department of Health, Australia) was used for this assay. Vero E6 (5 × 10^4^) cells were seeded in 96-well plates with DMEM medium, and incubated overnight at 37 °C and 5% CO_2_. After incubation, the medium was removed, heat inactivated mouse serum was serially diluted five-fold, and viral inoculum (~260 FFU/well) was incubated with serially diluted sera for 1 h at 37 °C. In a similar process to the mouse serum, a final concentration of 10 µg/mL of the nAb, S309 [28], was serially diluted five-fold, then incubated with similar amount of SARS-CoV-2 virus. The mixture (50 µL) was then added to each well of the cell plates to infect the cells. The overlay medium was added onto the cells, and the plates were re-incubated for 14 h at 37 °C and 5% CO_2_ prior to fixing the cells with 80% acetone. The plates were dried, blocked using blocking buffer for 1 h at room temperature. The block buffer contains milk diluent sera (KPL, Seracare) and 0.1% Tween in PBS. Plates were then probed with anti-spike antibody (CR3022) [29] and followed by IR dye^®^-conjugated 2^ry^ Abs (LI-COR Biosciences, Lincoln, NE, USA), both diluted in blocking buffer, were added to each of the seeded cell wells. The plates were read using an Odyssey Infrared Imaging System infrared high-resolution scanner LI-COR CLX (LI-COR Biosciences, Lincoln, NE, USA). Spots denoting the number of infected Vero E6 cells were counted using the procedure below.

VNT assay plate spots (signaling viral cell entry; Figure S2) were counted using ImageJ Fiji software version 1.53e (a free, open-source application, https://imagej.net/software/fiji/, accessed on 15 May 2021). The plate images were cropped, and the color threshold was adjusted to the default settings: black and white (B&W), in RGB color space with the parameters: Red = 33, Green = 74, and Blue = 49, against a dark background. The diluted serum wells across all plates were processed using the same parameters, including immune serum plates and the naïve serum plate. After threshold adjustment, we conducted particle counts for every well. The counts were divided by virus only (blank serum) positive control numbers to yield *N*% at a given dilution. For individual sera that exceeded 50% neutralization values at 1/20 or 1/40 dilutions, the reciprocal serum dilution that corresponded to 50% neutralization was interpolated to yield *N*_50_ values.

Correlation between VNT assay neutralization and cELISA using untreated individual mouse serum (R^2^ = 0.54) was much weaker compared to that of heat-inactivated individual sera (R^2^ = 0.975, *n* = 25) or combined group mean neutralization (R^2^ = 0.98, *n* = 10; Figure 4A–C). The overall weak-to-moderately neutralizing murine serum neutralization 50% efficacy values (*N*_50_) between both cELISA and VNT assays correlated very well (R^2^ = 0.94, *n* = 6) with the slope approaching unity (Figure 4D).

## 6. Assay Limitations

Our novel assay strategy is not limited to the detection of RBD-bound nAbs. *N*-terminus domain-bound nAbs can be determined by coating the plates with 100 µL of 3.32 µg/mL proline-substituted S-protein instead of RBD (21.23 kDa, on amino acid Mwt basis). This is an equimolar concentration to our assay RBD concentration, as S-protein monomer (141 kDa, on amino acid Mwt basis) contains a single RBD, while the trimer (423 kDa, on amino acid Mwt basis) contains three RBDs. An initial quick binding affinity titration may be required to establish the optimum S-protein coating concentration that would yield a suitable OD reading range for detection and quantification. A similar 6.6-fold increase in coating solution concentration using full-length S-protein, as substitute for RBD, was suggested in a recently published cELISA assay to yield comparable results to RBD-coated plates [6]. However, the limitation in all currently employed cELISA assays, including ours, is its lack of detection of priming process inhibitory Abs compared to cell-based assays. These Abs prevent furin and cathepsin-L enzymes from priming spike protein; however, they do not prevent RBD/ACE2 binding, so a different assay is required to evaluate this neutralization mechanism.

## 7. Reagents and Equipment

### 7.1. Reagents

- Recombinant SARS-CoV-2 spike-RBD-His (RBD, Sino Biological, Beijing, China, Cat. No. 40592-V08H);
- PBS tablets (Gibco, Carlsbad, CA, USA, Cat. No. 18912014);
- Tween 20 (Sigma-Aldrich, Victoria, Australia, Cat. No. P1379);
- Recombinant human ACE2 with human IgG-Fc (*hFc*-ACE2, Sino Biological, Beijing, China, Cat. No. 10108-H02H);
- Sodium hydrogen carbonate (Chem Supply, South Australia, Australia, Cat. No. SA001-500G);
- Sodium carbonate (Chem Supply, Cat. No. SA099-500G);
- Bovine serum albumin (BSA), heat shock fraction (Sigma-Aldrich, Cat. No. A7906-10G);
- Deionized water (resistivity ~18 MΩ cm);
- Sulfuric acid, 98% (Sigma-Aldrich, Cat. No. 258105)—CAUTION, sulfuric acid is a strong acid and must be handled inside of a fume hood;
- Secondary antibody (2^ry^ Ab): Peroxidase (HRP)-conjugated goat anti-human IgG-Fc Ab (Sigma-Aldrich, Cat. No. A0170);
- SigmaFast o-phenylenediamine dihydrochloride tablets (OPD, Sigma-Aldrich, Cat. No. P9187).

### 7.2. Equipment

- Polystyrene microtiter ELISA high affinity plates, 96 flat bottom wells (Sarstedt, South Australia, Australia, Cat. No. 82.1581.200);
- Magnetic bar and stirrer;
- Micropipettes;
- Auto-ELISA (Viaflo ASSIST, Integra Biosciences, Victoria, Australia) equipped with multichannel pipette (Biotools Pty Ltd., Queensland, Australia);
- Sterile pipette tips and Eppendorf tubes;
- Falcon tubes, 50 mL (BD, Cat. No. 352070);
- SpectraMax 250 microplate reader (Molecular Devices, San Jose, CA, USA);
- Originpro 2020 (OriginLab Corporation, Northampton, MA, USA) or Prism v8.3 software (GraphPad Software Inc., San Diego, CA, USA) for function fitting and data analysis;
- Airtight plastic storage box for ELISA microtiter plates.

### 7.3. Reagent Setup

Caution: Personal protective equipment (lab coat, goggles and acid-resistant gloves) should be worn, especially while handling sulfuric acid or OPD substrate. The reagent quantities required are as follows:

- *hFc*-ACE2 (12.5 µg/plate);
- RBD (5 µg/plate);
- Wash buffer, PBST (800 mL/plate);
- Bovine serum albumin (BSA) (0.675 g/plate);
- HRP-conjugated goat anti-human IgG-Fc Ab (3.3 µL/plate);
- OPD tablets (0.5 tablet/plate);
- Sulfuric acid (98%) (0.27 mL/plate).

#### Example Reagent Set-Up for Five Plates, including the Calibration Curve Plate

1. Prepare carbonate coating buffer (10 mL/plate):

Weigh out 193 mg sodium carbonate and 380 mg sodium hydrogen carbonate, then dissolve both in 100 mL of deionized water. Adjust the pH to 9.6, if necessary.

2. Prepare PBST wash buffer (800 mL/plate):

Add eight PBS tablets to 4 L of deionized water. Stir with a magnetic bar and stirrer, until the tablets are completely dissolved, then add 1 mL Tween 20 using a needleless 2 mL syringe.

3. Prepare 2% *w*/*v* BSA block solution (25 mL/plate):

Weigh out 5 g of BSA and dissolve this in 250 mL of PBST wash buffer.

4. Prepare 0.5% *w*/*v* BSA solution (35 mL/plate):

Weigh out 2.5 g of BSA and dissolve this in 500 mL of PBST wash buffer. Alternatively, transfer 125 mL of 2% *w*/*v* block solution and dilute to a final volume of 500 mL in a suitable glass bottle using PBST wash buffer.

5. Prepare *hFc*-ACE2 solution (10 mL/plate):

Within the supplier’s original glass vial, dissolve *hFc*-ACE2 in a suitable amount of deionized water to prepare 1 mg/mL solution. Transfer 125 µL (125 µg *hFc*-ACE2) into 50 mL 0.5% *w*/*v* BSA solution and mix well.

6. Prepare 2^ry^ Ab solution (10 mL/plate):

Warm the 2^ry^ Ab stock solution to room temperature, transfer 16.65 µL into 50 mL of 0.5% BSA solution and mix well.

7. Prepare OPD substrate (10 mL/plate) (prepare fresh before addition):

Dissolve three substrate buffer tablets in 60 mL of deionized water in a glass bottle using a magnetic bar and stirrer, until completely dissolved. Cover the bottle with foil to protect from light, then add three OPD substrate tablets and stir, until completely dissolved.

8. Prepare 1N sulfuric acid (10 mL/plate):

Transfer 1.35 mL of sulfuric acid (98%) to 20 mL of deionized water in a suitable glass bottle and complete volume to 50 mL with deionized water.

## 8. Assay Protocol

Experimental procedure takes about 4.5 + 8.5 h to complete and can be conducted over 2 days.

### 8.1. Plate Coating and Blocking (4.5 h)

**Figure 5:**
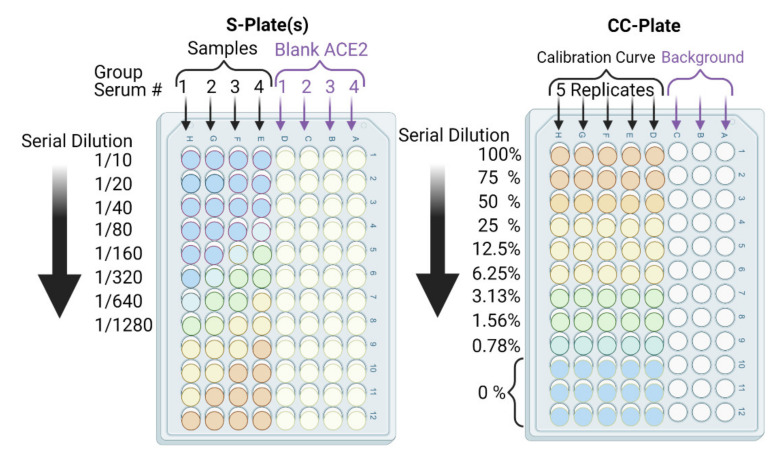
Schematic depicting cELISA plates: calibration curve (CC)-plate and sample (S)-plate arrangement, orientation, and associated dilutions. The lowest calibration curve value (0.78%) corresponds to our assay’s detection limit. Accurate quantitation starts from ≥6.25%. S-plate sample rows have RBD, BSA block, serum, ACE2, and 2^ry^ Ab. S-plate blank ACE2 rows have RBD, BSA block, the same serum, and 2^ry^ Ab. CC-plate calibration curve rows have RBD (different amounts), BSA block, ACE2, and 2^ry^ Ab. CC-plate background rows have BSA block, ACE2, and 2^ry^ Ab. To check non-specific serum interactions (with ACE2 and/or 2^ry^ Abs) and efficient washing (of ACE2 and 2^ry^ Abs), background and blank ACE2 rows are employed.

1. Dissolve RBD in a suitable volume of distilled water to prepare 1 mg/mL stock solution in a 1 mL Eppendorf tube.
2. Dilute RBD to a final concentration of 0.5 µg/mL RBD in carbonate buffer in a 50 mL Falcon tube, or other suitable container, by transferring the necessary volume from the stock solution prepared in Step 1.
3. Add a volume of 100 µL of RBD (0.5 µg/mL) in carbonate buffer to coat each well of the ELISA sample plates (S-plates), giving a total RBD amount of 50 ng/well.
4. In parallel, prepare a calibration curve plate (CC-plate) by adding 100 µL of, protein-free, carbonate buffer to five rows (D–H), starting from column 3, down to column 9. Add 25 µL of carbonate buffer to column 2 for five rows (D–H) (Figure 5).
5. Coat the wells of the CC-plate by adding 200 µL of the RBD solution prepared in Step 2 to column 1 for five rows (D–H), then conduct two-fold serial dilutions using a multichannel pipette by taking 100 µL from column 1 and transferring it to column 3. Mix the solution by pipetting 50 µL up and down five times, then transfer 100 µL to column 4, and repeat until you reach column 9. Discard the last 100 µL you withdraw from column 9. This should leave three blank rows (A–C) across all columns, as well as rows (D–H) in column 2 (Figure 5).

Critical: The volumes and concentration used in this step must be exact, as this will provide the calibration curve from which binding inhibition will be calculated.

6. Add 75 µL of the stock RBD solution (from Step 2) to the CC-plate column 2, rows D–H, using a multichannel pipette, then mix the solutions by pipetting 50 µL up and down five times.
7. Place the plates in an airtight storage container and incubate the S-plates and CC-plate for 90 min at 37 °C.
8. After incubation, dispose of the solution from the wells of the S-plates and CC-plate and wash the plates three times with deionized water, followed by three more washes with PBST wash buffer. Dry the plates by tapping upside down on paper towels.

The RBD amounts in the CC-plate will range from 50 ng to 0.097 ng per well. This covers neutralized-RBD from 0% (for 50 ng/well, column 1) to 99.3% (for 0.78 ng/well, column 9), including a 75% point. This provides a very practical and thorough range (Figure 5).

9. Block the CC-plate and S-plates by adding 250 µL of 2% bovine serum albumin (BSA) solution in PBST wash buffer to all wells, including the three empty rows (A–C) of the CC-plate, which provide background OD readings for substrate control (Figure 4).
10. Place the plates in an airtight storage container and incubate all plates overnight at 4 °C or for 90 min at 37 °C.
11. Repeat washing Step 8 (Pause Point).

### 8.2. Addition of Immune Murine Sera (3.5 h)

12. Add 180 µL of 0.5% BSA solution in PBST to the wells in column 1 of each S-plate, and 100 µL of the same solution to all other wells of the S-plates.
13. Conduct heat treatment on a 45 µL aliquot of undiluted/neat, vortexed mouse serum in an Eppendorf tube at 57 °C for 30 min using a temperature-controlled water bath.

Critical: This timing must be adhered to. Heating the serum for too long will inactivate the nAbs, while not heating for long enough will leave serum complement proteins active, resulting in non-specific binding interactions and obscured assay results.

14. Transfer 20 µL of each heat-treated serum sample to a designated well in column 1, e.g., well 1H, of the S-plate (Figure 5). Transfer another 20 µL of serum from the same sample to another well of column 1, e.g., well 1D, of the same S-plate (Figure 5). Thus, each serum sample is added twice, to two different wells, of column 1; the second well will serve as a blank ACE2 control.
15. After serum addition to the S-plates, mix the contents of the column 1 wells by pipetting up and down a 50 µL volume five times. Then, conduct a two-fold serial dilution using a multichannel pipette by transferring 100 µL from column 1, rows A–H, to column 2, rows A–H, and repeat until you reach column 12. Discard the last 100 µL you withdraw from column 12. Repeat Steps 14 and 15 for all S-plates.
16. Add 100 µL of 0.5% BSA solution to all wells of the CC-plate without further treatment.
17. Repeat incubation Step 7.
19. Repeat washing Step 8.

### 8.3. Addition of hFC-ACE2, 2^ry^ Ab and Substrate (5 h)

19. Add 100 µL of 2.5 µg/mL *hFc*-ACE2 solution to all wells of the CC-plate.
20. Add 100 µL of 2.5 µg/mL *hFc*-ACE2 solution to the E-H row wells of the S-plates only using a multichannel pipette, i.e., avoid the blank ACE2 control wells in rows A–D of the S-plates (Figure 5).

Critical: Be careful not to add ACE2 to the blank ACE2 controls: they are intended as a blank to subtract any background and minor cross-reactivity of the 2^ry^ Ab with murine sera.

21. Repeat incubation Step 7.
22. Repeat washing Step 8.
23. Dilute goat anti-human IgG-Fc 2^ry^ Ab to 1/3000 by adding 3.3 µL of 2^ry^ Ab stock to 10 mL of 0.5% BSA solution for each plate (3.3 µL/10 mL 0.5% BSA per plate).

Critical: Check the manufacturer’s instructions for 2^ry^ Ab dilution.

24. Add 100 µL of diluted 2^ry^ Ab solution to all wells of the S-plates and CC-plate.
25. Repeat incubation Step 7.
26. Repeat washing Step 8, and wipe underneath the plates dry using Kimwipes.

Critical: Washing must be thorough, as any residual 2^ry^ Ab, especially in the CC-plate, will obscure measurements.

27. Finally, add 100 µL of OPD substrate solution to all wells of the CC-plate and S-plates. Incubate the plates in the dark and allow them to react for 25 min at room temperature, then add a volume of 100 µL of stop solution, 1N sulfuric acid. Determine the absorbance at 450 nm OD_450_ using a common plate reader for all plates.

Critical: Be careful while using OPD substrate: it must be prepared fresh and kept in the dark once prepared and during reaction time. Otherwise, oxidation will result in a high background. Once established, proceed with full measurements (Pause Point).

### 8.4. Calculation Procedure (2 h)

The calculation sheet (GraphPad Prism project) is included in the Supplementary Materials to facilitate the calculation procedure of both CC- and S-plates.

**Figure 6:**
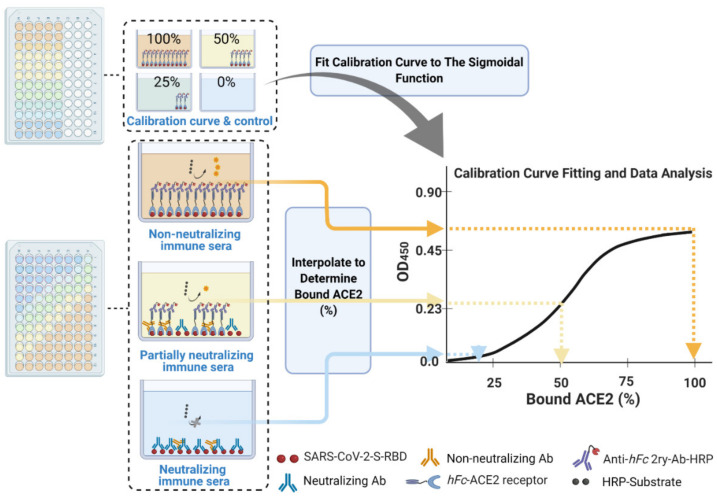
Schematic process diagram showing how to establish a calibration curve plot and interpolate the calibration curve using individual blank ACE2-corrected serum OD readings to obtain bound ACE2 (%) for each serum dilution.

28. Subtract the background OD_450_ (mean of the first three rows (A–C)) from individual calibration curve OD_450_ for each of the five rows (D–H) of each dilution (columns 1–9) to yield blank-subtracted calibration curve individual values (five values, rows D–H, for each dilution step, columns 1–9) (Figure 5).
29. Calculate the mean value (±standard deviation) of blank-subtracted calibration curve data and plot these against their associated concentration expressed in terms of bound ACE2 (%) (Figure 6), i.e., the OD reading average of the second and third columns correspond to the percent of available RBDs (37.5 and 25 ng, respectively) compared to the total RBD amount in column 1 (50 ng), 75% and 50% respectively.
  Critical: Check whether each point on the calibration curve (mean ± standard deviation) fits the following criteria:
  - Each point is significantly different from its neighboring points—this means a tight standard deviation is required. The mean and standard deviation OD_450_ values for 25%, 50%, 75%, and 100% bound ACE2 should all be significantly different (*p* < 0.05).
  - The lowest mean OD_450_ value is significantly higher than the background.
  - The highest mean OD_450_ value should be in the range of 0.5 to 1.0.
30. Fit the OD_450_ mean (±standard deviation) versus bound ACE2 (%) plot with a four-parameter logistic function (Figure 6) using Graphpad Prism or Originpro 2020 software using (Equation (1)), where y is the mean OD reading, A1 and A2 are the minimum and maximum mean OD_450_ readings, x is bound ACE2 (%), xo is bound ACE2 (%) at 50% OD_450_ signal, and p is the power exponent.

Critical: Check whether the logistic function is well-fit (R^2^ > 0.97).

31. Similarly, for the S-plates, subtract the OD_450_ of each serum’s blank ACE2 (from rows A–D) from the OD_450_ of the main serum (rows E–H) to yield corrected main serum OD_450_ data (the main serum placed in row H has its blank ACE2 serum sample in row D, see experimental procedure Steps 14–15), then subtract well D1 from H1, and D2 from H2 then D3 from H3, etc. This step aims to eliminate any 2^ry^ Ab cross-reactivity with murine sera or any non-specific interaction that positively contributed to the OD_450_ signal, including background.
  Critical: Check the following criteria:
  - Do any corrected main serum OD_450_ data points from the S-plate main sera exceed the sum of the 100% bound ACE2 calibration curve point? If several such points exist, then the heat treatment was not effective.
  - Does the OD_450_ of any of the blank ACE2 wells exceed 10% of the calibration curve’s 100% bound ACE2 OD_450_ value after blank subtraction? If high OD_450_ values are consistent across several wells, then the 2^ry^ Ab has high cross-reactivity to the serum employed. In this case, either switch to a less murine-cross-reactive 2^ry^ Ab, or switch to a biotinylated-ACE2 and streptavidin-HRP system. If the high values are found only on a few plate edges or sporadic wells, then it is more likely that the washing step was ineffective.
32. Interpolate each corrected main serum OD_450_ data point using the established calibration curve logistic fit equation. As the four parameters of the function have already been established (Step 30), substitute ‘y’ with each corrected main serum OD_450_ data point to determine the amount of bound ACE2 (%) at each dilution down the row of that serum, i.e., the corrected main serum OD_450_ data points from rows E–H and columns 1–12. This provides an accurate transformation of OD_450_ data into bound ACE2 (%) for each serum sample at each dilution step.

**Figure 7:**
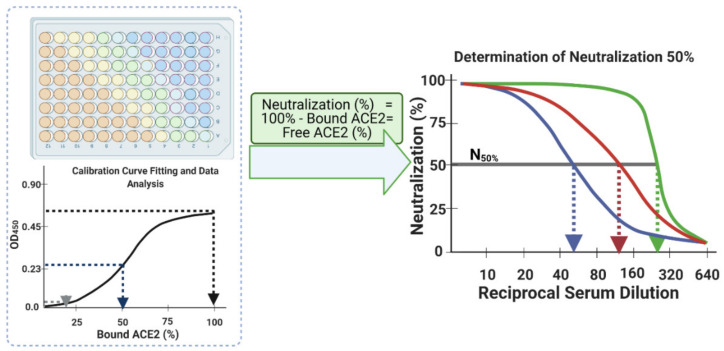
Schematic diagram depicting the processing of blank ACE2-corrected interpolated serum neutralization data from S-plates and the calibration curve (left panel), and plotting these against their corresponding reciprocal serum dilution values to obtain neutralization 50% (right panel dashed bold arrows) and nAb titers (right panel solid line thin arrows) for a given neutralizing serum sample.

Determine neutralization (%) from bound ACE2 (%) data points (for each serum sample at each dilution step) simply by subtracting the bound ACE2 (%) from 100% to yield neutralization (%) value at each dilution. This is equivalent to unbound ACE2 (%) or to blocked RBD (%) (Equation (2) and Figure 6 and Figure 7).

(2) $$\mathrm{Neutraliztion}\;\left(\%\right)=100\%-\mathrm{Bound}\;\mathrm{ACE}2\;\left(\%\right)$$

33. Finally, plot the neutralization (%) value for each serum sample in each well against its corresponding reciprocal serum dilution, as in Figure 5. The reciprocal serum dilutions of columns 1, 2, 3, 4, and 5 are 10, 20, 40, 80, 160, etc. Each curve represents the serum neutralization profile of different dilutions (Figure 7), and fit neutralization (%) versus corresponding reciprocal serum data (i.e., 1/serum dilution value, so 1/20 dilution becomes 20), again using the four-parameter logistic function (Figure 7).
34. Interpolate each serum curve at 50% neutralization value (Figure 7) to determine the neutralization 50% reciprocal serum dilution (*N*_50_ or *IC*_50_), other common determinant/endpoint of neutralization efficacy. The common endpoint/determinant of neutralization parameters (nAb titer, *N*_95_) can also be determined from the plot by interpolation at 95% neutralization; it is the maximum serum dilution value that results in 95% neutralization of ACE2 binding to coated RBD (Figure 7).
  - Standard nAbs can also be included and treated as serum, but without heat inactivation (Step 13). For comparison purposes, nAbs can be serially diluted from their stock solution until *N*_50_/*IC*_50_ is achieved. This also provides an additional external standard, as cell-based assay procedures often employ standard nAbs.
  - Interpolation at 80% or 90% neutralization yields *N*_80_ and *N*_90_ values, which are also commonly used neutralization efficacy endpoints.
  - If the results are not as expected, refer to troubleshooting section (Table 1).

## 9. Anticipated Results

**Figure 8:**
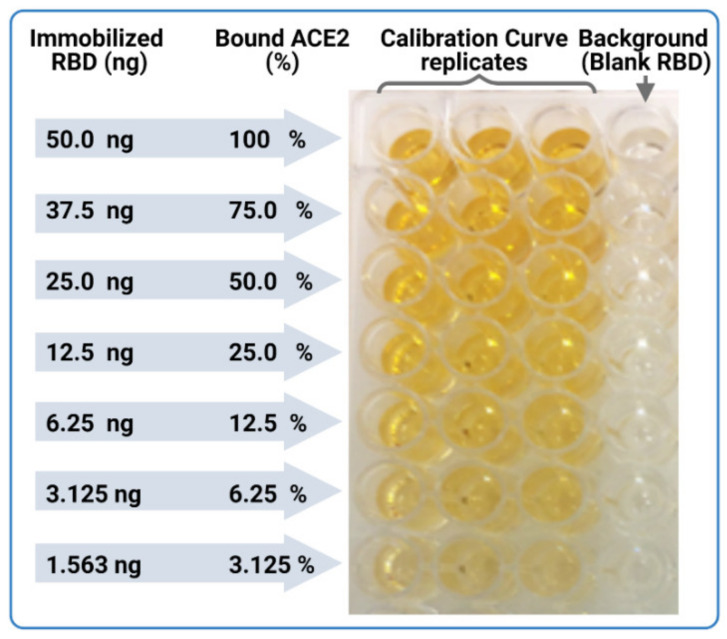
Sample of the calibration curve plate (CC-plate).

**Figure 9:**
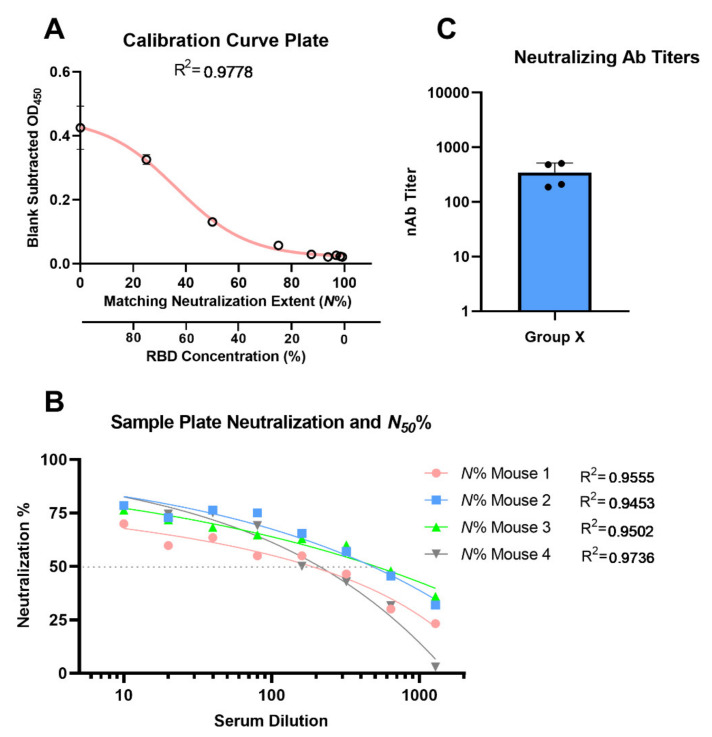
Depicting results of CC- and S-plate using the calculation sheet, fitting of calibration curve replicates to sigmoidal function (**A**), fitting of individual mice serum neutralization *N*% (of RBD-ACE2 binding) as a function of serum dilution (**B**), and the resulting mean group nAb titer column graph (**C**).

The calibration curve should be a gradient in intensity of substrate color, starting from intense color at high RBD concentration (column 1) to faded color (columns 8–10) (Figure 8). Visually, the background wells should be nearly transparent. The neutralizing sample sera should have the reverse intensity gradient of the CC-plates, starting from a fainter substrate color, changing to a more intense color, i.e., showing complete ACE2-RBD binding and absent neutralization in low serum dilution wells with consistent intense substrate color. The fitted calibration curve (OD-readings at various bound ACE2 concentrations) should match those in Figure 2. Example of using the auto-calculation sheet, in the Supplementary Materials, can also be found in Figure 9.

A calculation Sheet (GraphPad Prism v8.3 project: CELISA Calculation Sheet.pzfx) is included in the Supplementary Materials; the sheet has four data tables and three figures, which automatically calculates the neutralizing titers at *N*_50_. The first data table has editable yellow-colored cells for blank-corrected calibration curve OD_450_ readings (five replicates). The second data table has one S-plate editable yellow-colored cells for background-corrected four serially diluted sera, i.e., one S-plate. The rest of the calculation are conducted automatically by the calculation sheet, and the results include data and plots of neutralization *N*% for each individual serum at different dilutions, individual serum nAb titer at *N*_50_, and group-averaged nAb titer column graph (Figure 9).

## 10. Conclusions

This protocol offers a standardized and validated cELISA assay for the highly sensitive determination of neutralizing capacity of murine immune sera to support vaccine development against COVID-19. This protocol is of special interest as it presented the background of previously reported cELISA assays and pinpoints their shortcomings, thus ensuring standardization and optimization of key aspects of the assay. Moreover, we discussed future strategies for the development of similar assays to determine the human sera neutralization, against original or mutant variants of SARS-CoV-2. Furthermore, we have also demonstrated that the protocol method efficacy is equivalent to gold standard assays, such as VNT assay, while surpassing method validation criteria. This renders the protocol method suitable as a high-throughput in vitro efficacy assay, without physical containment requirements, which supports the progress of vaccine development.

## Figures and Tables

**Table 1:** Troubleshooting.

Issue	Potential Reason(s)	Solution
Neutralizing extent exceeded the plate dilution down the rows	Very highly neutralizing serum, *N*_50_ exceeding 2 × 10^4^.	Repeat the test starting with a lower serum dilution, e.g., 1/100, or conduct three-fold serum serial dilutions instead of two-fold.
Neutralization unapparent at the highest serum concentration	Irrelevant or non-neutralizing serum sample. Excessive heat treatment.	Repeat the assay with careful monitoring of heat treatment conditions (time and water bath temperature) and include a known standard murine neutralizing antibody (nAb) as an external standard to check assay integrity.
OD_450_ of blank ACE2 wells consistently exceeds 10% of the calibration curve’s 100% bound OD_450_ signal	Cross-reactivity of secondary antibody (2^ry^ Ab) with murine sera.	Employ a more species-specific 2^ry^ Ab with lower cross-reactivity or switch to the biotinylated ACE2 and streptavidin-HRP system
High blank ACE2 well OD_450_ in plate-edge or sporadic wells	Ineffective washing step.	Ensure you thoroughly wash every well in the plate three times with deionized water and three times with wash buffer. If feasible, use an automatic plate washer.
S-plate column 1, main serum well OD_450_ exceed the sum of blank ACE2 OD_450_ and 100% bound ACE2 OD_450_ combined	Heat treatment was not effective. Serum proteins are non-specifically interacting with ACE2 and 2^ry^ Abs at high serum concertation.	Repeat the assay with careful monitoring of heat treatment conditions (time and water bath temperature). Highly neutralizing serum data points (above 2% serum concentrations; S-plates, columns 1–3) can be excluded and neutralization efficacy calculations can still be conducted.
Non-significant differences between calibration curve OD_450_ values for 25%, 50%, 75%, or 100% bound ACE2	Variable amounts or coating times between wells of the CC-plate, resulting in high standard deviations of a given bound ACE2 (%) point. OD_450_ are all too high or too low, refer to reasons mentioned below.	Repeat the assay. Be consistent and quick with CC-plate coating. Incubate overnight at 4 °C for coating RBD, if necessary. Make sure the multichannel pipette is performing well and all seals are intact, or employ auto-ELISA equipment. If OD_450_ values are too high or too low, refer to solutions below.
The lowest calibration curve mean OD_450_ (0.8% bound ACE2 point) is equal to the background	A different, less sensitive substrate type was used, or the substrate was defective. Low substrate concentration or low 2^ry^ Ab concertation.	Conduct a quick experiment using only the CC-plate to adjust the 2^ry^ Ab and substrate. (1) Concentrate 2^ry^ Ab following the manufacturer’s specifications at a higher value than employed, but within the recommended stated range. (2) Adjust the substrate concentration and reaction time.
Highest mean OD_450_ value is ≫1.0.	A different, more sensitive substrate type, e.g., TMB, was employed. High 2^ry^ Ab concentration. OD_450_ > 1.0 will further convolute the calibration curve non-linearity.	Conduct a quick experiment using only the CC-plate to adjust 2^ry^ Ab and the substrate. (1) Dilute the 2^ry^ Ab following the manufacturer’s specifications at a lower value than employed, but within the recommended stated range. (2) Adjust the substrate concentration and reaction time.

## Data Availability

Not applicable.

## Supplementary Materials

The following are available online at https://www.mdpi.com/article/10.3390/vaccines9121493/s1, Figure S1: S309 neutralizing antibody VNT neutralization profile at different dilutions, logistic fit, and determined VNT *N*_50_ value; Figure S2: Example cell-based VNT neutralization assay plates with immune murine sera. GraphPad Prism v8.3 project: CELISA Calculation Sheet.pzfx.

## References

[1] Wen W., Su W., Tang H., Le W., Zhang X., Zheng Y., Liu X., Xie L., Li J., Ye J. (2020). Immune cell profiling of COVID-19 patients in the recovery stage by single-cell sequencing. Cell Discov..

[2] Hoffmann M., Kleine-Weber H., Pöhlmann S. (2020). A Multibasic Cleavage Site in the Spike Protein of SARS-CoV-2 Is Essential for Infection of Human Lung Cells. Mol. Cell.

[3] Lan J., Ge J., Yu J., Shan S., Zhou H., Fan S., Zhang Q., Shi X., Wang Q., Zhang L. (2020). Structure of the SARS-CoV-2 spike receptor-binding domain bound to the ACE2 receptor. Nature.

[4] Shang J., Ye G., Shi K., Wan Y., Luo C., Aihara H., Geng Q., Auerbach A., Li F. (2020). Structural basis of receptor recognition by SARS-CoV-2. Nature.

[5] Shalash A.O., Hussein W.M., Skwarczynski M., Toth I. (2021). Key Considerations for the Development of Safe and Effective SARS-CoV-2 Subunit Vaccine: A Peptide-Based Vaccine Alternative. Adv. Sci..

[6] Abe K.T., Li Z., Samson R., Samavarchi-Tehrani P., Valcourt E.J., Wood H. (2020). A simple protein-based surrogate neutralization assay for SARS-CoV-2. JCI Insight.

[7] Chen L., Liu B., Sun P., Wang W., Luo S., Zhang W., Yang Y., Wang Z., Lin J., Chen P.R. (2020). Severe Acute Respiratory Syndrome Coronavirus-2 Spike Protein Nanogel as a Pro-Antigen Strategy with Enhanced Protective Immune Responses. Small.

[8] Pandey M., Ozberk V., Eskandari S., Shalash A.O., Joyce M.A., Saffran H.A., Day C.J., Lepletier A., Spillings B.L., Mills J.L. (2021). Antibodies to neutralising epitopes synergistically block the interaction of the receptor-binding domain of SARS-CoV-2 to ACE 2. Clin. Transl. Immunol..

[9] Charbonneau R. (1991). New test for AIDS. IDRC Rep..

[10] Butler J.E. (1992). The behavior of antigens and antibodies immobilized on a solid phase. Struct. Antigens.

[11] Crowther J.R. (2000). Systems in ELISA. The ELISA Guidebook.

[12] Padoan A., Bonfante F., Pagliari M., Bortolami A., Negrini D., Zuin S., Bozzato D., Cosma C., Sciacovelli L., Plebani M. (2020). Analytical and clinical performances of five immunoassays for the detection of SARS-CoV-2 antibodies in comparison with neutralization activity. EBioMedicine.

[13] Mercado N.B., Zahn R., Wegmann F., Loos C., Chandrashekar A., Yu J., Liu J., Peter L., McMahan K., Tostanoski L.H. (2020). Single-shot Ad26 vaccine protects against SARS-CoV-2 in rhesus macaques. Nature.

[14] Koishi A.C., Suzukawa A.A., Zanluca C., Camacho D.E., Comach G., Dos Santos C.N.D. (2018). Development and evaluation of a novel high-throughput image-based fluorescent neutralization test for detection of Zika virus infection. PLoS Negl. Trop. Dis..

[15] Whiteman M.C., Bogardus L., Giacone D.G., Rubinstein L.J., Antonello J.M., Sun D., Daijogo S., Gurney K.B. (2018). Virus Reduction Neutralization Test: A Single-Cell Imaging High-Throughput Virus Neutralization Assay for Dengue. Am. J. Trop. Med. Hyg..

[16] Von Rhein C., Scholz T., Henss L., Kronstein-Wiedemann R., Schwarz T., Rodionov R.N., Corman V.M., Tonn T., Schnierle B.S. (2021). Comparison of potency assays to assess SARS-CoV-2 neutralizing antibody capacity in COVID-19 convalescent plasma. J. Virol. Methods.

[17] Tan C.W., Bogardus L., Giacone D.G., Rubinstein L.J., Antonello J.M., Sun D., Daijogo S., Gurney K.B. (2020). A SARS-CoV-2 surrogate virus neutralization test based on antibody-mediated blockage of ACE2–spike protein–protein interaction. Nat. Biotechnol..

[18] He Y., Zhu Q., Liu S., Zhou Y., Yang B., Li J., Jiang S. (2005). Identification of a critical neutralization determinant of severe acute respiratory syndrome (SARS)-associated coronavirus: Importance for designing SARS vaccines. Virology.

[19] Yu M., Stevens V., Berry J.D., Crameri G., McEachern J., Tu C., Shi Z., Liang G., Weingartl H., Cardosa J. (2008). Determination and application of immunodominant regions of SARS coronavirus spike and nucleocapsid proteins recognized by sera from different animal species. J. Immunol. Methods.

[20] Zhang F., Yu M., Weiland E., Morrissy C., Zhang N., Westbury H., Wang L.-F. (2006). Characterization of epitopes for neutralizing monoclonal antibodies to classical swine fever virus E2 and Erns using phage-displayed random peptide library. Arch. Virol..

[21] Huang Y., Zhao R., Luo J., Xiong S., Shangguan D., Zhang H., Liu G., Chen Y. (2008). Design, synthesis and screening of antisense peptide based combinatorial peptide libraries towards an aromatic region of SARS-CoV. J. Mol. Recognit..

[22] Wang Q., Zhang L., Kuwahara K., Li L., Liu Z., Li T., Zhu H., Liu J., Xu Y., Xie J. (2016). Immunodominant SARS Coronavirus Epitopes in Humans Elicited both Enhancing and Neutralizing Effects on Infection in Non-human Primates. ACS Infect. Dis..

[23] Stevens F.J., Bobrovnik S.A. (2007). Deconvolution of antibody affinities and concentrations by non-linear regression analysis of competitive ELISA data. J. Immunol. Methods.

[24] Sadana A., Vo-Dinh T. (1997). Antibody-antigen binding kinetics. A model for multivalency antibodies for large antigen systems. Appl. Biochem. Biotechnol..

[25] Walker S.N., Chokkalingam N., Reuschel E.L., Purwar M., Xu Z., Gary E.N., Kim K.Y., Helble M., Schultheis K., Walters J. (2020). SARS-CoV-2 Assays to Detect Functional Antibody Responses That Block ACE2 Recognition in Vaccinated Animals and Infected Patients. J. Clin. Microbiol..

[26] Amarilla A.A., Modhiran N., Setoh Y.X., Peng N.Y., Sng J.D., Liang B., McMillan C.L., Freney M.E., Cheung S.T., Chappell K.J. (2021). An Optimized High-Throughput Immuno-Plaque Assay for SARS-CoV-2. Front. Microbiol..

[27] Watterson D., Wijesundara D.K., Modhiran N., Mordant F.L., Li Z., Avumegah M.S., McMillan C.L., Lackenby J., Guilfoyle K., van Amerongen G. (2021). Preclinical development of a molecular clamp-stabilised subunit vaccine for severe acute respiratory syndrome coronavirus 2. Clin. Transl. Immunol..

[28] Pinto D., Park Y.J., Beltramello M., Walls A.C., Tortorici M.A., Bianchi S., Jaconi S., Culap K., Zatta F., De Marco A. (2020). Cross-neutralization of SARS-CoV-2 by a human monoclonal SARS-CoV antibody. Nature.

[29] Wrobel A.G., Benton D.J., Hussain S., Harvey R., Martin S.R., Roustan C., Rosenthal P.B., Skehel J.J., Gamblin S.J. (2020). Antibody-mediated disruption of the SARS-CoV-2 spike glycoprotein. Nat. Commun..

