# Supplementary material for: Detection and Quantification of SARS-CoV-2 Receptor Binding Domain Neutralization by a Sensitive Competitive ELISA Assay
Source: Vaccines (Basel). 2021 Dec 16;9(12):1493. doi: 10.3390/vaccines9121493 (PMC8705285; doi:10.3390/vaccines9121493)
Supplement: Supplementary file 1 [file vaccines-09-01493-s001.zip › vaccines-1487253-supplementary.pdf]

vaccines

MDPI

Protocol

Supporting Information

Detection and Quantification of SARS-CoV-2 Receptor Binding Domain Neutralization by a Sensitive Competitive ELISA Assay

Ahmed O. Shalash[1,*], Armira Azuar[1], Harrison Y. R. Madge[1], Naphak Modhiran[1,4], Alberto A. Amarilla[1], Benjamin Liang[1], Alexander Khromykh[1], Daniel Watterson[1,4], Paul R. Young[1,4], Istvan Toth[1,2,3], and Mariusz Skwarczynski[1,*]

[1] School of Chemistry and Molecular Biosciences, the University of Queensland, St. Lucia, QLD 4072, Australia.
[2] Institute for Molecular Bioscience, the University of Queensland, St. Lucia, QLD 4072, Australia.
[3] School of Pharmacy, the University of Queensland, Woolloongabba, QLD 4102, Australia.
[4] Australian Institute for Bioengineering and Nanotechnology, the University of Queensland, St. Lucia, QLD 4072, Australia
* Correspondence: M.S.: m.skwarczynski@uq.edu.au and A.S.: a.shalash@uq.edu.au

Citation: Shalash, A.O.; Azuar, A.; Madge, H.Y.R.; Modhiran, N.; Amarilla, A.A.; Liang, B.; Khromykh, A.A.; Watterson, D.; Young, P.R.; Toth, I.; et al. Detection and Quantification of SARS-CoV-2 Receptor Binding Domain Neutralization by a Sensitive Competitive ELISA Assay. *Vaccines* **2021**, *9*, 1493. https://doi.org/10.3390/vaccines9121493

Academic Editors: Soo-Hong Lee, Hansoo Park, Jagathesh Chandra Rajendran and K.S Jaganathan

Received: 15 November 2021
Accepted: 14 December 2021
Published: 16 December 2021

Publisher's Note: MDPI stays neutral with regard to jurisdictional claims in published maps and institutional affiliations.

Copyright: © 2021 by the authors. Licensee MDPI, Basel, Switzerland. This article is an open access article distributed under the terms and conditions of the Creative Commons Attribution (CC BY) license (http://creativecommons.org/licenses/by/4.0/).

Standard neutralizing antibody (nAb), S309, was employed in the VNT assay as an external standard. This potent nAb binds to RBD-derived K444T epitope [28]. The inhibi tion profile with serially diluted nAb concentrations was fitted to a sigmoidal logistic function and IC50 was derived (Figure S1). The maximum neutralizing expressed as percent reduction in the number of spots in the VNT assay compared to the number of spots of the positive control, i.e. uninhibited virus only cell entry. The determined IC50 or N50 values agreed with the reported neutralization value of about 20 ng/mL [28] against SARS-CoV-2. VNT assay plates with green spots signaled viral cell entry (Figure S2).

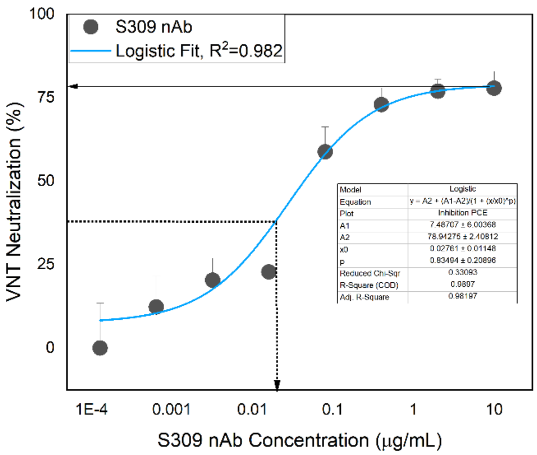

Figure S1. S309 neutralizing antibody VNT neutralization profile at different dilutions, logistic fit, and determined VNT N_{50} value.

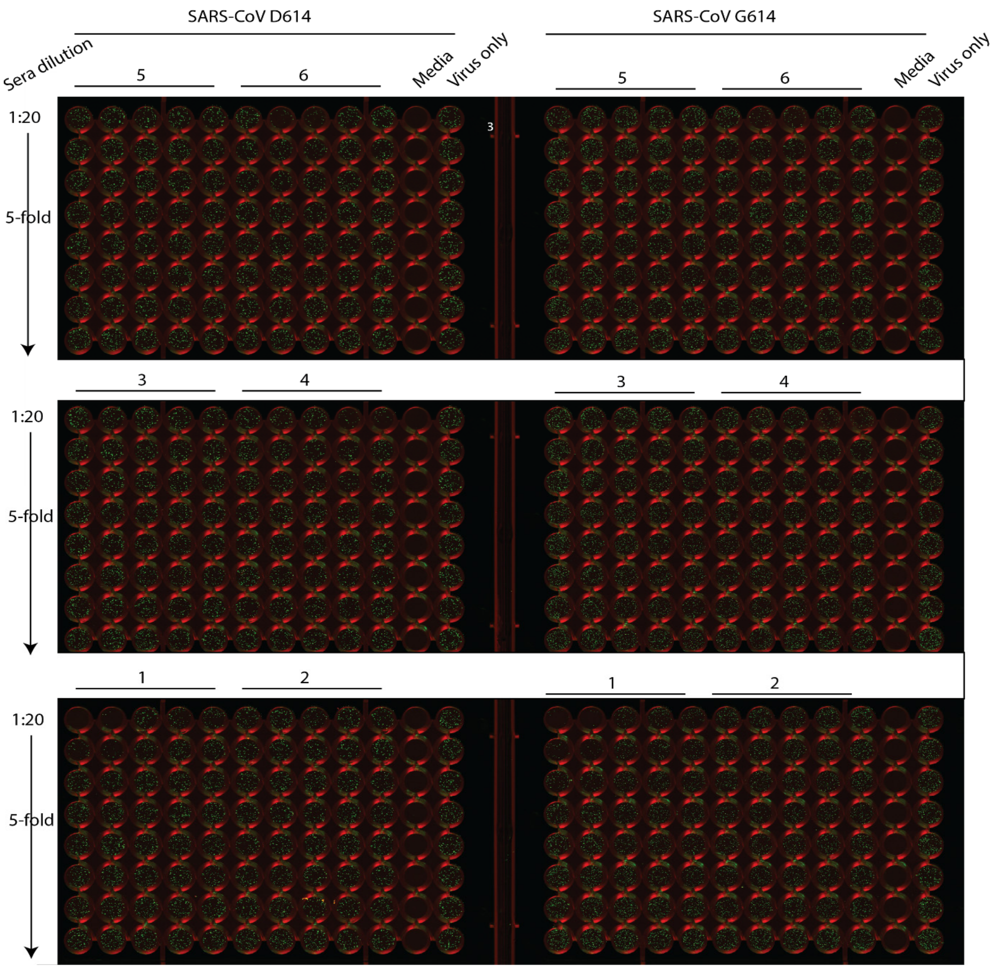

Figure S2. Example cell-based VNT neutralization assay plates with immune murine sera.
